# Dens Sana in Corpore Sano

**Published:** 1913-02

**Authors:** Wm. Brady

**Affiliations:** 1008 Lake Street, Elmira, N. Y.


### Dens Sana in Corpore Sano

By WM. BRADY, M.D.
1008 Lake Street, Elmira, N. Y.

IT is a long step in evolution from the low browed, heavy jowled aborigine with a full complement of molar machinery to the modern brain worker with scant room in his mandible for the eruption of the wisdom teeth. In the lower races and in atavistic types of our own race the jaw protrudes beyond the perpendicular brow line; in the Caucasian race the jaw is on the perpendicular, and in degeneratives the jaw frequently falls behind the perpendicular. Civilization varies as the square of the jaw—with a few old roots thrown in.

One or even all four of the third molars are missing from the jaws of forty-seven per cent. of adult Americans, and the lateral incisor is missing in six per cent. Civilized man no longer burdens his digestion with the crude materials supplied by Nature for the building of strong teeth. Impacted molars, troublesome wisdom teeth, dental caries, and interstitial gingivitis are Nature's expressions of protest against the refinements of our modern diet.

Two essential elements in the building and conservation of the teeth are lime and phosphorus. The source of these elements is largely vegetable. The cereals, fruits and certain green vegetables which can be eaten raw furnish calcuim, phosphorus, iron, potassium, magnesium, silicon and chlorine in the most assimilable form.

The "Staff of Life" was so named long before the days of refined white flour. The apology for bread now in popular use does not furnish the blood, bone and tooth making material which health demands. The mills of Minneapolis grind slowly but they grind exceedingly fine—fine enough to remove the layer of mineral food from the wheat kernel and leave little more than devitalized starch. Our daily bread leads us into constipation but it does not deliver us from anaemia and carious teeth. While awaiting the advent of more Horace Fletchers crying in the wilderness we should teach our patients to compensate the deficiency of mineral matter—the mineral starvation, by eating freely of raw cabbage, lettuce, onions, celery and fruits, articles of diet which also effect a saving in tooth brushes.

Oral sepsis from carious teeth is so common that we are apt to overlook its far reaching influences upon the general economy. How much tuberculosis results from carious teeth we can but surmise. We know that many children are infected via the cervical lymph nodes as a direct sequence of neglected oral hygiene, and we know that the bronchial glands may subsequently become involved in such cases.

The relation between septic states of the mouth and disease of the tonsils and adenoid tissue of the pharynx is a subject of debate. The stomatologists attribute the primary role to the carious teeth, while the laryngologists insist that the dental caries is induced by infection from the diseased tonsils. Adenoid hypertrophies likewise cause or are caused by defective dental and palatal development. In some instances removal of the adenoids and tonsils fails to overcome the nasal stenosis unless the dentist works in conjunction with the physician; a jack-screw appliance inserted and worn, under the dentist's care, for several weeks, spreads the palate arch, gradually brings it down, permits nasal expansion and thus prevents the recurrence of the adenoid tumor.

The entire subject of oral hygiene is beautifully summed up in Goadbey's recent Hunterian lecture:

"Lowered vitality favors the vegetation of microorganisms in the mouth; these attack the teeth, and the diseased teeth still further lower vitality so that a vicious circle is established."

It is the physician's duty to attack a vicious circle from every corner, if I may so put it. A simple anaemia, sometimes perhaps a pernicious anaemia, requires not only hematinics but also diet and treatment directed toward the frequently present carious teeth or pyorrhoea alveolaris. An atrophic gastritis or an intestinal autotoxemia very commonly has its origin in the mouth and will not radically improve without due attention to oral sepsis. I have never seen a case of chronic rheumatoid or metobolic arthritis that did not also present a chronic gingivitis, and I believe that the same chronic toxemia may explain certain cases of chronic nephritis.

Oral hygiene is too often neglected in surgical practice. It is entirely logical to attribute certain post-operative pneumonias to a faulty technique in which the surgeon forgets to disinfect the septic mouth preceding anaesthesia, and so preserves a handy culture of virulent penumococci to enliven the after history. The development of tonsillitis following a nasal operation is clinical evidence of faulty antisepsis of the oral cavity. The operator depends too much upon a simple mouth wash or gargle to clean up the old culture of pneumococci universally present in the mouth.

Talbot, who has done much research work on oral sepsis, states that dental caries may be prevented in children by painting the gums once a month with a solution of zinc iodid fifteen, water ten, iodin twenty-five, and glycerine fifty parts. If we can preserve the temporary teeth by such simple means, it will not be necessary for posterity to place the permanent set in a glass of water beside the Seidlitz powders every night. As a personal asset a sound tooth is better than a gold crown, and a whole mouthful of good dental organs will gain for a man almost anything he desires in this country, unless it be a permanent berth in the White House.

The average practitioner scarcely realizes the importance of the mouth as a microbe factory. We know something of the diphtheria carrier, and we know something of the pneumococcus carrier, but we do not lay sufficient stress on the need of dental treatment in routine work. A periostitis or a purulent sinus elsewhere in the body invariably excites our interest, but the same conditions in the oral cavity, the most active absorbent area imaginable, are neglected merely because they are so common. This practice may have been proper enough in the old days when family doctors pooh-poohed anything less than a serious fit of sickness, but it cannot be justified any longer. The modern trend of medical science is in the direction of prophylaxis, and phophylaxis naturally begins in the mouth.

Young children should be encouraged to masticate crusts, coarse vegetables, raw fruits and the like as soon as they cut their milk teeth. The baby should be allowed to gnaw at a bone whenever he is so inclined. The schoolboy should be allowed to crack nuts with his teeth to his heart's content. These habits give nourishment and exercise to the developing teeth and jaws.

In the evolution of the mandible, the result of dietetic selection, we find an explanation for the increasing prevalence of pyorrhoea alveolaris or interstitial gingivitis.

Oral hygiene means conservation of the teeth and a great deal more. I am sure I have witnessed general improvement of the health of children who receive good dental care at the free clinic established by the Elmira Dental Society. And I have repeatedly "diagnosed" a dental cavity from the noticeable disturbance of general health that caries invariably produces in my own children. The reason we do not recognise the intimate relation between decayed teeth and physical deficiency is that we do not have the opportunity to observe the sequence of symptoms intimately, as one has in his own household.

The race should return to whole wheat bread. The profession should preach the doctrine of Mr. Horace Fletcher and consistently follow the preaching by prescribing the correct diet. At least two great mills are marketing a whole wheat flour that makes a very palatable bread. We have the reason, we have the means; all that remains for us to do is to prescribe. The last word in oral prophylaxis is Fletcherism.

